# Novel Gel Microemulsion as Topical Drug Delivery System for Curcumin in Dermatocosmetics

**DOI:** 10.3390/pharmaceutics13040505

**Published:** 2021-04-07

**Authors:** Cristina Scomoroscenco, Mircea Teodorescu, Adina Raducan, Miruna Stan, Sorina Nicoleta Voicu, Bodgan Trica, Claudia Mihaela Ninciuleanu, Cristina Lavinia Nistor, Catalin Ionut Mihaescu, Cristian Petcu, Ludmila Otilia Cinteza

**Affiliations:** 1 Polymer Department, National Institute for Research and Development in Chemistry and Petrochemistry-ICECHIM, 202 Spl. Independentei, 060021 Bucharest, Romania; scomoroscencocristina@gmail.com (C.S.); trica.bogdan@gmail.com (B.T.); claudia.ninciuleanu@yahoo.com (C.M.N.); lc_nistor@yahoo.com (C.L.N.); mihaescu_catalin96@yahoo.com (C.I.M.); 2 Faculty of Applied Chemistry and Materials Science, University Politehnica of Bucharest, 010737 Bucharest, Romania; mircea.teodorescu@upb.ro; 3 Physical Chemistry Department, University of Bucharest, 030018 Bucharest, Romania; adina.raducan@g.unibuc.ro; 4 Department of Biochemistry and Molecular Biology, Faculty of Biology, ICUB-Research Institute of the University of Bucharest, University of Bucharest, 050095 Bucharest, Romania; miruna.stan@bio.unibuc.ro (M.S.); sorina.voicu@bio.unibuc.ro (S.N.V.)

**Keywords:** curcumin, gel microemulsion, transdermal delivery, microemulsion, dermatocosmetics, grape seed oil

## Abstract

Gel microemulsion combines the advantages of the microemulsion, which can encapsulate, protect and deliver large quantities of active ingredients, and the gel, which is so appreciated in the cosmetic industry. This study aimed to develop and characterize new gel microemulsions suitable for topical cosmetic applications, using grape seed oil as the oily phase, which is often employed in pharmaceuticals, especially in cosmetics. The optimized microemulsion was formulated using Tween 80 and Plurol^®^ Diisostearique CG as a surfactant mix and ethanol as a co-solvent. Three different water-soluble polymers were selected in order to increase the viscosity of the microemulsion: Carbopol^®^ 980 NF, chitosan, and sodium hyaluronate salt. All used ingredients are safe, biocompatible and biodegradable. Curcumin was chosen as a model drug. The obtained systems were physico-chemically characterized by means of electrical conductivity, dynamic light scattering, polarized microscopy and rheometric measurements. Evaluation of the cytotoxicity was accomplished by MTT assay. In the final phase of the study, the release behavior of Curcumin from the optimized microemulsion and two gel microemulsions was evaluated. Additionally, mathematical models were applied to establish the kinetic release mechanism. The obtained gel microemulsions could be effective systems for incorporation and controlled release of the hydrophobic active ingredients.

## 1. Introduction

Microemulsions are appreciated in the cosmetic industry due to their transparency, stability and minimal energy consumption as a result of spontaneous formation when the ratios of oil, water and surfactant mix are appropriate [1]. These colloidal vectors have many applications, but in particular, they are used as systems that can encapsulate and deliver large amounts of hydrophilic and hydrophobic active ingredients [2,3]. Another important advantage of microemulsions is the ability to protect the encapsulated active pharmaceutical ingredients (API) from degradative reactions [4,5].

Many active ingredients have been incorporated into microemulsions that form the basis for cosmetic or pharmaceutical formulations, including Deepaline PVB (palmitoyl hydrolyzed wheat protein), for its wrinkle-reducing effect [6]; Alpha-tocopherol (alpha-T), which is a powerful antioxidant [7]; azelaic acid, an active ingredient used in anti-acne treatments [8]; Vitamin A palmitate [9]; babchi essential oil, with antipsoriatic effect [7]; neem oil, with strong antibacterial effect; beta-carotene [10], the combination of arbutin with lactic acid and niacinamide, which together have a strong whitening effect [8].

Some of these cosmetic colloidal systems are already commercially available [11] in personal care products such as shampoos, conditioners, deodorants, sunscreens, and moisturizers. A requirement that must be fulfilled by the microemulsions is the biocompatibility of each component [12]. A disadvantage of these colloidal systems is the high concentration of surfactant or mixtures of surfactants, which can cause skin irritation, but the use of surfactants, accepted in the cosmetics industry, that are less aggressive, biocompatible, and have low irritant potential, represents a solution [13]. Microemulsions are also characterized by low viscosity, which is another disadvantage for most topical cosmetic formulations [13]. The addition of a viscosity-increasing agent to the microemulsion system leads to the formation of a gel microemulsion, a system that takes advantage of the microemulsion and the gel at the same time.

Gels are highly appreciated in the personal care industry [11]. By converting the microemulsion into a gel microemulsion, the contact of the product with the skin and the release period of the active ingredient are prolonged [3,14]. Thus, the gel microemulsion has an attractive texture and pleasant consistency, is simple to apply, and ensures prolonged release of cosmetic assets [15]. Due to the high content of surfactant mixtures, microemulsions are able to facilitate skin penetration and ensure a constant and controlled release, instead of the burst release of cosmetic active ingredients.

Curcumin, the main compound of the turmeric rhizome (Curcuma longa L. Family: Zingiberaceae), is appreciated for its anti-oxidant, anti-inflammatory, wound healing and anti-microbial characteristics [16,17,18]. With so many benefits for the human body, especially for the skin, Curcumin is used in a variety of cosmetics and pharmaceutical applications [19].

In the present work, we aimed to prepare and characterize new gel microemulsions suitable for topical applications in dermatocosmetics, using grape seed oil as the oily phase, and a mixture of cosmetic surfactants and biocompatible polymers. The study also aimed to determine the ability to encapsulate Curcumin in the systems obtained for the first time, both in the microemulsion and in the gel microemulsions. The selected oily phase, grape seed oil, is a biocompatible ingredient that itself contains cosmetic active ingredients, and it is often used in pharmaceuticals and, especially, cosmetics.

In this study, a mixture of biocompatible surfactants, at a minimum concentration, but which ensures the stability of the system, was chosen. To obtain the gel microemulsions, three biocompatible polymers, one synthetic and two polymers of natural origin, were selected. Carbopol^®^ 980 NF was selected as a synthetic gelling agent, because it is biocompatible and non-toxic [20]. The first natural polymer selected was the sodium salt of hyaluronic acid, which, especially at a high molecular weight, can increase viscosity, being also one of the primary structural components of the dermis and epidermis, with the specification that with age, the amount of hyaluronic acid in the epidermis decreases considerably or even disappears [21,22,23]. In addition to sodium hyaluronate, high molecular weight chitosan was the second natural polymer selected. It is an amino-polysaccharide derived from chitin, appreciated for its biocompatibility, bacteriostatic effect and wound-healing properties [23].

The current trend in dermatocosmetics is to formulate products with oils from natural sources, e.g., vegetable oils, with intrinsic therapeutic activity. However, obtaining Winsor IV microemulsions with vegetable oils is still considered a real challenge. Additionally, the restrictions in the cosmetics industry regarding surfactants must be taken into account. In this work, the prepared formulations contain a vegetable oil as the oily phase; moreover, the surfactants and polymers used were selected only from among ingredients approved in the cosmetics industry. To the best of our knowledge, this is the first study that reports the preparation and characterization of gel-type microemulsions, based on grape seed oil, Plurol^®^ Diisostearique CG, Tween 80, ethanol, water and polymers. The microemulsion with selected composition (denoted LVM-low viscosity microemulsion) was characterized, and the ability to encapsulate Curcumin in this system was analyzed by high-performance reverse-phase liquid chromatography (HPLC). Additionally, the composition of the gel microemulsion is unique and proves the possibility of obtaining suitable changes in viscosity using hyaluronic acid and chitosan as natural polymers with beneficial anti-aging activity in cosmetic formulations. The LVM microemulsion was converted into gel microemulsions by incorporating the biocompatible polymers listed above at different concentrations, which were studied in terms of the ability to control the release of the Curcumin as a model active ingredient and compared with the polymer-free (simple) microemulsion.

## 2. Materials and Methods

### 2.1. Materials

Cold-pressed grape seed oil (MAYAM Cosmetics, Oradea, Romania), Tween 80 and ethanol absolute (SIGMA-ALDRICH), Plurol^®^ Diisostearique CG (Polyglyceryl-3 Diisostearate—PDCG, kindly gifted by Gattefosse, Saint-Priest, France), Carbopol^®^ 980 NF (high molecular weight poly(acrylic acid), Lubrizol Advanced Materials, Inc, Wickliffe, OH, USA), hyaluronic acid sodium salt (Streptococcus Equi sp., molecular weight ~1.5–1.8 × 10^6^ Da, SIGMA-ALDRICH), chitosan (highly viscous, Fluka^®^), Curcumin (95%, Natures aid), MTT (3-(4,5-dimethylthiazol-2-yl)-2,5-diphenyltetrazolium 1-bromide (97.50%, Sigma-Aldrich) and human keratinocyte cells (HaCaT, an immortal keratinocytes cell line spontaneously transformed from adult human skin, Cell Lines Service GmbH, Eppelheim, Germany) were used as received. Acetonitrile (>99.9%), phosphoric acid solution (>85%) and ethanol (>99.8%) solvents, used for HPLC analysis, were purchased from Sigma-Adrich (Sigma-Adrich Chemie GmbH, Taufkirchen, Germany) and were all HPLC grade purity. Distilled water was employed in all experiments.

### 2.2. Preparation of the Microemulsion and Gel Microemulsions

To obtain the single-phase optimized microemulsion, grape seed oil, Tween 80, Plurol^®^ Diisostearique CG, ethanol and distilled water in certain ratios were stirred at 2500 rpm by using a Vortex equipment for 1 min and the obtained mixture was left to equilibrate for 24–48 h at 25 °C. Single-phase microemulsions were prepared by stirring the suitable amount of surfactants in oily and aqueous phases and left to stand for at least 24 h, since equilibrium is not immediately achieved in the systems with vegetable oils and surfactants. For the incorporation of Curcumin, a prolonged mixing time is needed to obtain complete dissolution of the active ingredient and to achieve the equilibrium of the loaded microemulsion due to the very different solubility of Curcumin in each component.

The preparation of gel microemulsions using Carbopol^®^ 980 NF and hyaluronic acid sodium salt was carried out by adding the polymer to the previously prepared LVM microemulsion under magnetic stirring, for 24–48 h until complete dissolution of the polymer, as previously described. The polymer concentrations in the gel microemulsions, expressed as mass percentage based on the entire microemulsion mass, are displayed in Table 1.

To achieve the maximum viscosity, 5% NaOH solution was added to the Carbopol gel microemulsions. The preparation of gel microemulsions using chitosan was made in two steps. In the first step, chitosan was dissolved into 1% acetic acid to obtain solutions of 0.5%, 1%, 1.3%, and 1.5% polymer concentration. Next, gel microemulsions of various polymer concentrations were obtained by adding the other microemulsion components to the chitosan solutions previously prepared, in the same ratio as above and stirring.

The selection of the polymer concentrations was made based on the rheological characteristics. A small concentration does not necessarily offer viscosity, but at higher concentrations the polymers could not be completely solubilized in the LVM microemulsion. Therefore, a range of suitable concentrations was selected, in order to obtain the desired viscosity and also to allow comparison between the rheological characteristics of different polymers at the same concentrations.

### 2.3. Incorporation of Curcumin

Curcumin incorporation was carried out by adding it to the LVM microemulsion or gel microemulsions and magnetically stirring the mixture for 24–48 h until complete dissolution. To determine the maximum encapsulation capacity, surplus Curcumin was added to some samples, followed by centrifugation at 13,000 rpm for 10 min to remove the excess [24].

### 2.4. Incorporation Capacity of the Microemulsion

Reversed-phase high-performance liquid chromatography (HPLC) was employed to determine the incorporation ability of Curcumin in the microemulsion and selected gel microemulsions using a system made up of a Jasco LC-NetII/ADC HPLC equipped with a Jasco PU-2089Plus Quaternary Gradient Pump, a Jasco UV–2075Plus Intelligent UV–VIS detector, and a Teknokroma HPLC NUCLEOSIL 100 C 18 5 μm 25 × 0.4 column. The mobile phase was composed of 50 vol.% acetonitrile and 50 vol.% of a 1 vol.% phosphoric acid solution and the flow rate was set at 1 mL/min [25]. The elution of Curcumin was monitored at 420 nm [17]. All samples were diluted in a 1:200 mass ratio with ethanol before analysis. The standard calibration curve was obtained by employing samples of 0.5 wt.%; 0.75 wt.% and 1 wt.% Curcumin in the corresponding microemulsion. The resulted equation of the calibration curve was y = 65.4x + 6.2833, with a correlation coefficient R² = 0.9989.

### 2.5. Characterization of Microemulsions and Gel Microemulsions

The microemulsion droplet size was measured using dynamic light scattering (DLS) (Nano ZS ZEN3600 Zetasizer, Malvern Instruments, Malvern, UK) equipment. Electrical conductivity was tested in triplicate on undiluted samples using a Cole-Parmer conductivity meter model 500. The microemulsion was analyzed using cross-polarized light microscopy (Optica Microscopes Instruments, Ponteranica, Italy), equipped with a Levenhuk M1400 PLUS Microscope video camera to collect digital images. A small quantity of microemulsion was placed on the glass slide and covered with a plastic coverslip and the picture was taken at a 10× magnification, at 25 °C. The rheological properties of the microemulsion and gel microemulsions were determined using a Kinexus Pro rheometer (Malvern Instruments, UK) with 1.60 software for data acquisition, equipped with a Peltier element for temperature control. The measurements were carried out in rotation mode at 25 °C using a 1°/40 mm cone. Each sample was equilibrated for 5 min before measurement.

### 2.6. In Vitro Release of Curcumin

The release behavior of Curcumin from the LVM microemulsion and the microemulsions gel H4LVM and CT4LVM was determined by in vitro release experiments, at 25 °C, by using a Spectra/Por™ (Spectrum™, New Jersey, NJ, USA) regenerated cellulose dialysis membrane tubing of 6.4 mm diameter, with 12.000–14.000 Dalton MWCO. The dialysis membrane was previously hydrated for 24 h. A certain amount of each sample was placed into the dialysis bag and immersed into the receptor medium that completely covers the sample. The receptor medium consisting of a 1:1 (*v/v*) water/ethanol mixture [26] was placed under magnetic stirring at 200 rpm. After certain time intervals, 1 mL samples of the receptor medium were withdrawn and replenished with fresh receptor medium, maintaining a constant volume. The amount of released Curcumin was analyzed using the HPLC method, described above. The results were expressed as percent of cumulative drug release, using the formula: CR% = the cumulative release amount of Curcumin/the total amount of Curcumin in the formulation × 100 [27].

### 2.7. In Vitro Permeation Study

In vitro study for Curcumin skin permeation was performed using a vertical Franz cell (PermeGear, Inc., Hellertown, PA, USA). The effective diffusion area was 0.99 cm^2^. The 8 mL receptor compartment was filled with medium consisting of a 1:1 (*v/v*) phosphate-buffered saline (PBS; pH 7.4)/ethanol mixture [28]. The diffusion cell was maintained at 37 °C and the receptor chamber was continuously homogenized using a magnetic stirrer. Each experiment was performed in triplicate. Strat-M^®^ membrane, a synthetic membrane that is predictive of diffusion in human skin [27], was used for the study. The membrane does not need to be hydrated prior to use and was loaded into the Franz cell, with the shiny layer upward [28]. Next, 0.5 g of the microemulsion was added to the donor chamber, and the donor was sealed with Parafilm^®^ to prevent sample evaporation. After certain time intervals (2, 3, 4, 5, 6, 24 h), 0.5 mL samples of the receptor medium were withdrawn and replenished with a fresh receptor medium to maintain constant volume. Additionally, the Curcumin retained in the model membrane was measured. At the end of each experiment, the membrane was carefully cleaned, in order to remove the excess sample. The part of the membrane exposed to the sample was cut and soaked in 5 mL of ethanol and stirred for 30 min at 37 °C. The permeation samples were analyzed by using the HPLC method, described above. The standard calibration curve was obtained by employing samples from 0.001 wt.% to 0.015 wt.% (eight points) Curcumin in the 50% ethanol solution. The calibration curve equation was found to be y = 1.5498x − 5.8829, with a correlation coefficient R² = 0.9944.

Experimental data were used to determine the cumulative Curcumin permeation per unit of membrane surface area (*Q_t_*), the steady-state permeation flux (*J_ss_*) and apparent permeability coefficients (*P_app_*), calculated using equations from the literature [28]. The cumulative Curcumin permeation *Q_t_* (expressed as cumulative drug permeation per unit of model membrane area) was calculated using Equation (1):

(1) $$Q_{t}=V_{r}C_{t}+\overset{t-1}{\underset{i=0}{\sum }}V_{s}C_{i}$$

where *C_t_* is the drug concentration in the receiver media (ethanolic solution 50%) at the selected sampling time t, *C_i_* is drug concentration of the i-th sample, and *V_r_* and *V_s_* are the volumes of the receiver solution and the sample, respectively.

The steady-state fluxes (*J_ss_*) were calculated by linear regression interpolation of the experimental data at a steady state, according to Equation (2):

(2) $$J_{ss}=\frac{∆Q_{t}}{\left(∆t\times S\right)}$$

Apparent permeability coefficients (*P_app_*) were calculated using Equation (3):

(3) $$P_{app}=\frac{J_{ss}}{C_{d}}$$

where *C_d_* is Curcumin concentration in the microemulsion donor compartment. The experiment was performed to ensure sink conditions, thus presuming that the drug concentration in the receiver solution is negligible compared to that in the donor compartment.

### 2.8. Biocompatibility

Cell viability was determined by the MTT test, which is a colorimetric test that analyzes cell metabolism and is based on the reduction of the MTT compound under the action of mitochondrial succinate dehydrogenases (NAD (P) H-dependent mitochondrial dehydrogenase succinate) present in the metabolically active cells. The reduction of yellow soluble tetrazolium salts from the MTT and the formation of violet formazan crystals is indicative of the presence of viable cells. Evaluation of the cytotoxicity by the MTT test allows the quantification of metabolic activity directly related to the number of living cells. Human keratinocyte cells were cultured in DMEM culture medium (Dulbecco’s modified Eagle Medium) supplemented with 2 mM l-glutamine, 100 units per milliliter of antibiotic mix (Penicillin/Streptomycin/Amphotericin) and 10% fetal bovine serum, and kept in an incubator humidified at 37 °C with a CO_2_ concentration of 5%.

To evaluate the cytotoxic effect of the samples (LVM, CP3LVM, H4LVM, CT4LVM) on HaCaT cells, each sample was added to sterile 24-well culture plates, at a concentration of 1 µL sample per 1 mL culture medium. Immediately after the addition of the samples, culture medium was added with cells at a seeding density of 2∙× 10^5^ cells per well of 1.9 cm^2^. Incubation of the cells with samples was performed in an incubator at 37 °C for 24 h. Untreated cell wells were used as controls. Next, the examination was performed under the inverted optical microscope in phase contrast Olympus IX71 (Olympus, Tokyo, Japan) with a 10× objective. The culture medium was immediately removed and washed with 1 mL phosphate-buffered saline (PBS) per well and 500 μL of MTT solution (1 mg/mL) was added to each well. After two hours of incubation with the MTT solution, it was removed, and the formazan crystals are solubilized with isopropanol. At the end of the procedure, cell viability was determined spectrophotometrically at a wavelength of 595 nm using the FlexStation 3 Multi-Mode board reader (Molecular Devices, San Jose, CA, USA).

## 3. Results

### 3.1. Preparation and Characterization of the Optimized Microemulsion for Curcumin Incorporation

To obtain the optimized microemulsion, preliminary studies were performed to determine the optimal concentrations and ratios for each ingredient for the water system–grape seed oil–mix of surfactants, using the pair of surfactants Tween 80 and PDCG.

As the oily phase is a vegetable oil, high concentrations—mass concentrations of about 80%—of surfactants are required to obtain a WIV-type microemulsion. In a previous paper [29], we developed a microemulsion with grape seed oil as an oily phase and Tween 80 and 1-octanol as the surfactant and co-surfactant, respectively. Winsor IV-type microemulsions were obtained, with a concentration of the surfactant–*co*-surfactant mix of around 70%, and low viscosity, at below 0.25 Pa∙s, that was capable of encapsulating a significant amount of Curcumin. However, taking into account the restrictions on surfactant concentrations in the pharmaceutical and cosmetic fields, the possibility of obtaining microemulsions with lower concentrations of surfactant mixture by adding ethanol co-solvent was studied. Systems with oil–water ratios of 2:1 and 3:1 were further investigated, since for the 1:1 ratio, no single-phase microemulsions were obtained, even at high surfactant mixture concentrations. The ratio 3:1 was finally selected, in order to facilitate the solubilization of the polymer and formation of gel microemulsion systems. Winsor IV microemulsions were obtained for the Plurol-Tween 80 mixture only at very high concentrations, usually above 70%, depending on the surfactant–*co*-surfactant ratio. For the Plurol-Tween 80 mixture at a 1:3 ratio, it was possible to obtain a single-phase microemulsion at a minimum surfactant mixture concentration of 63% with the aid of ethanol as cosolvent. The microemulsion (called LVM) with the minimum surfactant content was selected, and the ingredients of the microemulsion, their function and concentration are shown in Table 2. The visual appearance of the LVM microemulsion was examined over 1 year at a temperature of 25 °C, and the lack of change confirmed its stability over time.

**Figure 1:**
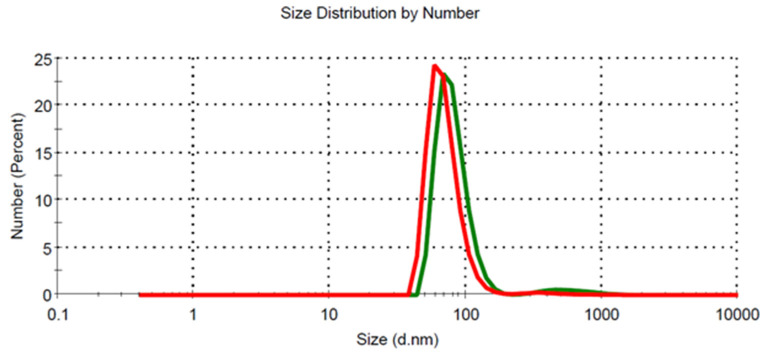
Size and size distribution of undiluted and freshly prepared microemulsion without Curcumin (red) and with Curcumin (green).

To obtain information about the structural state, the LVM microemulsion was characterized by electrical conductivity measurements, DLS, in order to determine the size of the discontinuous phase droplets, and polarized light microscopy. The conductivity measurements provided a value of electrical conductivity of 228 μS/cm, which is characteristic for an O/W type microemulsion [30,31,32,33]. The DLS measurements showed that the discontinuous phase droplets had a uniform dimensional distribution and an average diameter of about 74.73 ± 6.06 nm (Figure 1). The encapsulation of drugs did not produce significant changes in the microemulsion droplet, and the average size was found to be 75.52 ± 5.22 nm.

The stability of the optimized microemulsion with and without encapsulated Curcumin was also investigated by measuring of size and size distribution of droplets after one year storage in the absence of light, under refrigeration. As expected, the microemulsion was stable over a long period of time, and the average diameter of droplets was found to be 71.44 ± 2.08 nm for the void microemulsion and 72.56 ± 3.38 nm for the microemulsion with Curcumin. Additionally, the encapsulated active ingredient Curcumin exhibited stability in terms of solubility in the colloidal carrier. No visible micro-crystals of the drug were observed after storage.

The LVM sample was examined using a polarized light microscope in order to differentiate between the microemulsion and the lyotropic liquid crystal phase [34]. The polarized light microscopy images obtained for the investigated sample did not show birefringence, and no texture could be observed in the polarized light, so according to the literature, the sample can be classified as a microemulsion and it can be stated that it does not contain lyotropic liquid crystal phases [35]. Based on the composition, visual appearance of the microemulsion, conductivity and polarized light microscopy, the microemulsion was classified as a WIV-type O/W microemulsion.

### 3.2. Physico-Chemical Characterization of Gel Microemulsions

Microemulsions are characterized by a low viscosity, which is not suitable for topical applications [26,36]. Gel microemulsions, which are characterized by higher viscosity, are considered to be a beneficial alternative. The selection of thickeners is made according to the type of the continuous phase of the formulation, which can be aqueous or oily [37]. Because the LVM sample was classified as an O/W microemulsion, three types of water-soluble polymers were selected for the preparation of gel microemulsions, one synthetic (Carbopol^®^ 980 NF) and two natural (hyaluronic acid sodium salt and chitosan).

**Figure 2:**
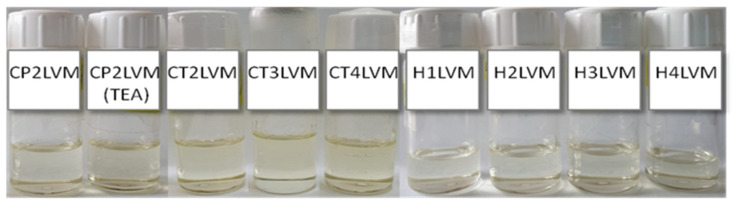
Visual appearance of gel microemulsions used as Curcumin delivery system.

**Figure 3:**
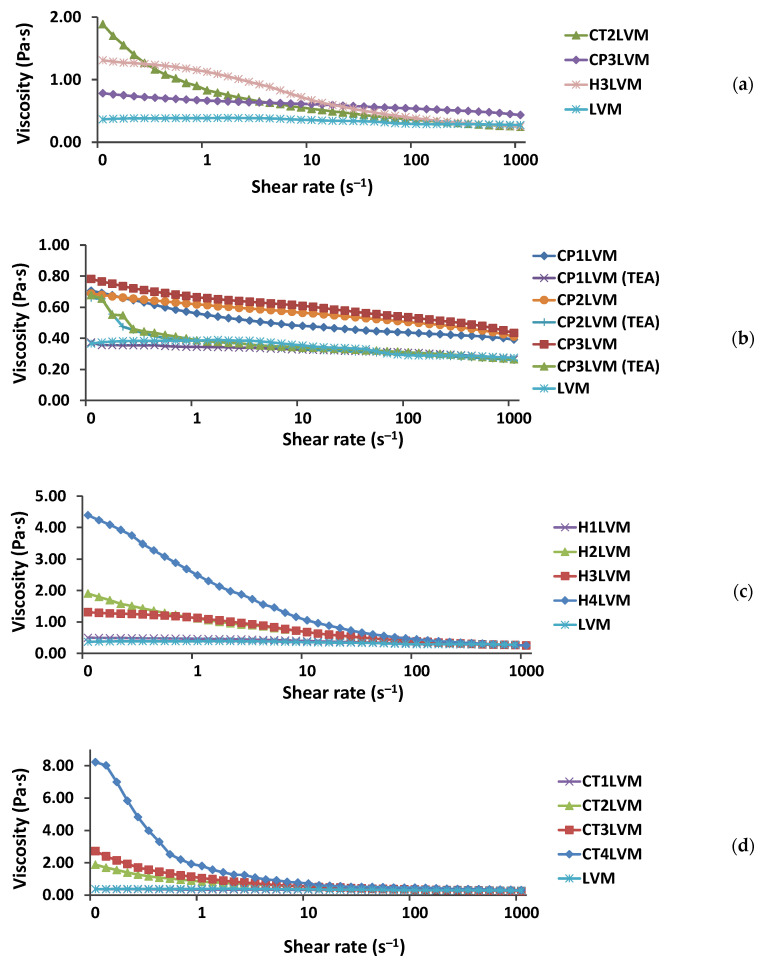
Flow curves for LVM microemulsion and gel microemulsions obtained with: (**a**) mass concentration of approximately 0.2% thickening agent; (**b**) Carbopol^®^ 980 NF (“TEA” for gel microemulsions neutralized with triethanolamine); (**c**) hyaluronic acid salt; (**d**) chitosan.

The prepared gel microemulsions were homogeneous and transparent (Figure 2), and were characterized in terms of rheological behavior compared to the LVM microemulsion (Figure 3). The LVM microemulsion showed a Newtonian behavior, its viscosity being about 0.36 Pa∙s. The relatively low value of the viscosity was due to the presence of a low concentration of oil simultaneously with a high concentration of water [37].

The obtained gel microemulsions showed a pseudoplastic non-Newtonian rheological behavior, with decreasing viscosity with increasing shear rate (Figure 3).

A similar mass concentration of the thickening agent (approximately 0.2%), Carbopol^®^ 980 NF, generated a significantly lower viscosity value compared to the other two polymers, for which relatively close viscosity values were obtained (Figure 3a).

Additionally, the increase in the concentration of Carbopol^®^ 980 NF (Figure 3b) produced a much smaller increase in the viscosity of the microemulsion compared to sodium hyaluronate (Figure 3c) and chitosan (Figure 3d). This behavior can be explained by the lower solubility and globule conformation of the poly (acrylic acid) in the aqueous phase of the microemulsion.

According to the manufacturer, “the sample containing the Carbopol^®^ 980 NF polymer must be neutralized until a pH of 6–7 is reached, to obtain a maximum viscosity” [38]. Neutralization of poly (acrylic acid) leads to an increase in its solubility in water and the adoption of a more relaxed conformation, which increases the viscosity of the solution.

To increase the viscosity of the microemulsion in the presence of Carbopol^®^ 980 NF, two types of neutralizing agents were tested in this study, namely a 5% NaOH solution and triethanolamine, respectively. In fact, when the pH reached 6–7, the viscosity of the system decreased in both cases and flocs/precipitate appeared. This can be explained by the presence of ethanol in the microemulsion system, which reduces the solubility of Carbopol^®^ 980 NF in the continuous phase, following its transformation into sodium, or, respectively, triethylammonium salt. For this reason, microemulsions with Carbopol were abandoned.

### 3.3. Incorporation Efficiency of the Microemulsion and Gel Microemulsions

The solubility of Curcumin in water is extremely low, but it is higher in oils, co-solvents and surfactants [39,40]. For this reason, we can affirm that, in the obtained system of microemulsion and gel microemulsions, Curcumin was incorporated in the oily phase of the microemulsion and in the surfactant film formed at the oil–water interface.

Reverse-phase liquid chromatography (HPLC) was used to quantify the incorporation capacity of the prepared microemulsions for Curcumin [24,41]. The analysis of Curcumin showed a chromatogram with three peaks, corresponding, respectively, to Curcumin (the majority compound, 70%), demethoxycurcumin (20%) and bisdemethoxycurcumin (10%) [42], with the last two compounds representing impurities [41]. The results obtained were consistent with data from the literature [17,42].

The calibration curve was constructed by representing the total area of the peaks as a function on the concentration of solubilized Curcumin (representing Curcumin with impurities) in the LVM microemulsion. To avoid changes in the appearance of the chromatogram caused by the components of the microemulsion, Curcumin encapsulated in the microemulsion system was used as a standard.

**Figure 4:**
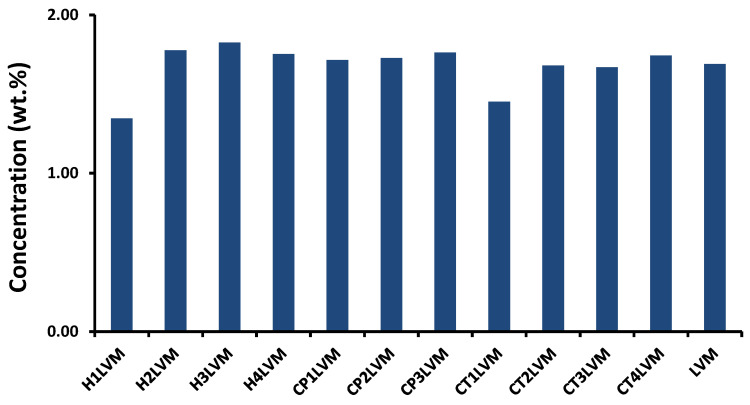
The incorporation efficiency of Curcumin with variation of the polymer content in gel microemulsion: H—for LVM with sodium hyaluronate; CP—for LVM with Carbopol; CT—for LVM with chitosan. For details on sample content, see Table 1.

To determine the maximum incorporation capacity, an excess of Curcumin was added to the chosen systems [39], and after saturation of the microemulsion, the excess Curcumin was separated by centrifugation. A Curcumin-loaded microemulsion sample was then injected into HPLC. Based on the previously determined calibration curve, the maximum amount of Curcumin that can be solubilized in LVM microemulsion and gel microemulsions was identified (Figure 4). The maximum Curcumin capacity that could be encapsulated in the LVM microemulsion (1.69 wt.%, Figure 4) was approximately 2-fold higher than in the microemulsion prepared with oleic acid, Smix (ethanol and Tween 80, Span 80, n-butanol) and water, as described by S. Sharma et al. [43]. In the case of gel microemulsions, there was generally a very small increase in the incorporation capacity of Curcumin (Figure 4) compared to simple microemulsion, probably due to the interaction between drug molecules and polyelectrolyte chains. This increase may also be due to the two impurities of the active principle, which had a more hydrophilic character than pure Curcumin [17] and a higher compatibility with polymers, which also have a hydrophilic character. In the case of samples H1LVM and CT1LVM, the gel microemulsions formulated with the smallest amounts of the polymers hyaluronic acid and chitosan, the incorporation capacity slightly decreased. The molecular interaction between the Curcumin and the two polymers in such reduced concentrations was not studied, and further research is needed to explain it.

According to data from the literature, the solubility of Curcumin in water is very low, almost insignificant [44]; the solubility of Curcumin in oil increases slightly [45], but in microemulsion and gel microemulsion systems, it is possible to solubilize a significant amount of Curcumin. The incorporation capacity of gel microemulsions did not change considerably depending on the nature and concentration of the polymer used, which can be explained by the different localization phases of the two components: the polymer in the aqueous phase, and Curcumin in the oily phase.

### 3.4. In Vitro Release of Curcumin

To select the most suitable formulation for topical application, it is necessary to establish the amount of Curcumin that is released from the gel microemulsion system and to analyze the release profile [46]. The release of Curcumin was evaluated at 25 °C, using a regenerated cellulose dialysis membrane and a 1:1 (*v/v*) water/ethanol mixture, considered in the literature as a suitable release medium [29,47,48], wherein Curcumin is soluble. To study the properties of controlled release, the gel microemulsion systems with the highest viscosity were chosen, namely the H4LVM, CT4LVM samples, whose release behavior was compared with the control system, namely the LVM microemulsion.

**Figure 5:**
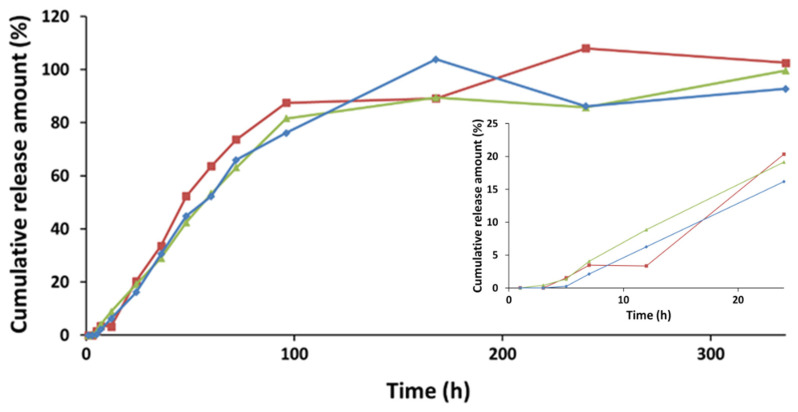
Curcumin release profile of LVM microemulsion (red), 0.3 wt.% sodium hyaluronate in LVM (blue), and 0.34 wt.% Chitosan in LVM (green)**.**

Figure 5 displays the curves for the cumulative amount of Curcumin released from the three microemulsion systems. For all systems, an initial induction period of approximately three hours was observed (Figure 5 inset), followed by a constant increase in the amount of Curcumin released. Thus, most of the Curcumin was released in up to 100 h, approximately 80%, followed by a flattening of the release curve up to 336 h. A possible explanation for the behavior in the first three hours could be the existence of an induction period, due to the different composition of the continuous phase of the microemulsion, i.e., water, and the release medium made up of 1:1 *v/v* water/ethanol, respectively. In microemulsions, Curcumin located in the oil droplets and the surfactant film formed at the oil–water interface, being insoluble in the continuous phase, cannot diffuse outside the membrane, or the diffusion rate was extremely low. After placing the microemulsion in contact with the release medium, ethanol from the release medium penetrated the membrane due to the different concentration and, gradually, the ethanol concentration in the microemulsion continuous phase increased until the concentrations on both sides of the dialysis membrane became equal. With the increase of the ethanol concentration in the microemulsion continuous phase, the solubility of Curcumin in this phase increased, and thus it began to be released from the oil droplets and from the oil–water interface, thus reaching the release medium. As the ethanol concentration in the microemulsion continuous phase increased, the rate of Curcumin release progressively increased as well, until the ethanol/water ratio in the continuous phase and the release medium were equalized. After that, a constant Curcumin release rate was first noticed, which decreased slightly over time. Based on the shape of the release curves, we estimate that the equalization of the water/ethanol ratio occurred after three hours from the beginning of the experiment for all systems (Figure 5 inset).

It can also be observed that by increasing the viscosity of the microemulsions, namely the formation of gel microemulsions, the release profile of the cosmetic active ingredients was not significantly influenced. Compared to other microemulsions that are employed as release systems for Curcumin and show an over 70% release in less than 24 h [39,49,50,51], the three systems obtained in this study display a much slower release of Curcumin, therefore making it possible to obtain a prolonged release. This feature is essential in cosmetic applications, where a gradual and prolonged release of active principles is desired.

To evaluate the release profile of the Curcumin from the selected formulations, the experimental data were fitted to equations describing different release kinetics. The following mathematical models were used: zero-order, first-order, Higuchi and Korsmeyer-Peppas. The zero-order model refers to the process of slow and constant drug release from a drug delivery system, where the release of the active substance is independent of the initial drug concentration. The first-order model characterizes a release process where the amount of drug released at any time is directly proportional to the drug concentration. The Higuchi model supposes a homogeneously dispersed drug that is released from the delivery system through a pure diffusion release mechanism, without erosion or swelling of the matrix occurring [52]. The Korsmeyer-Peppas model describes the drug release from a polymeric delivery system. In this model, the release profile depends on the diffusion exponent “*n*”, namely, *n* = 0.5 suggests a Fickian diffusion; 0.45 < *n* < 1.0 means a non-Fickian transport (both diffusional and relaxational transport); for *n* = 1.0, non-Fickian case-II drug transport mechanism is characteristic, meaning the zero-order model, while for *n* > 1, the non-Fickian super case-II drug transport mechanism is characteristic [53,54,55].

Because the number of parameters was not identical for each model, and R^2^ increases with increasing numbers of parameters, for the selection of the mathematical model which fits best on the experimental data, it was necessary to use the adjusted correlation coefficient (R^2^_adj_) [56,57]. This formula was used: R^2^_adjusted_ = 1 − ((*n* − 1) × (1 − R2))/(*n*p − 1), where “p” is the number of parameters in the model and “*n*” is the number of experimental data points [58].

**Figure 6:**
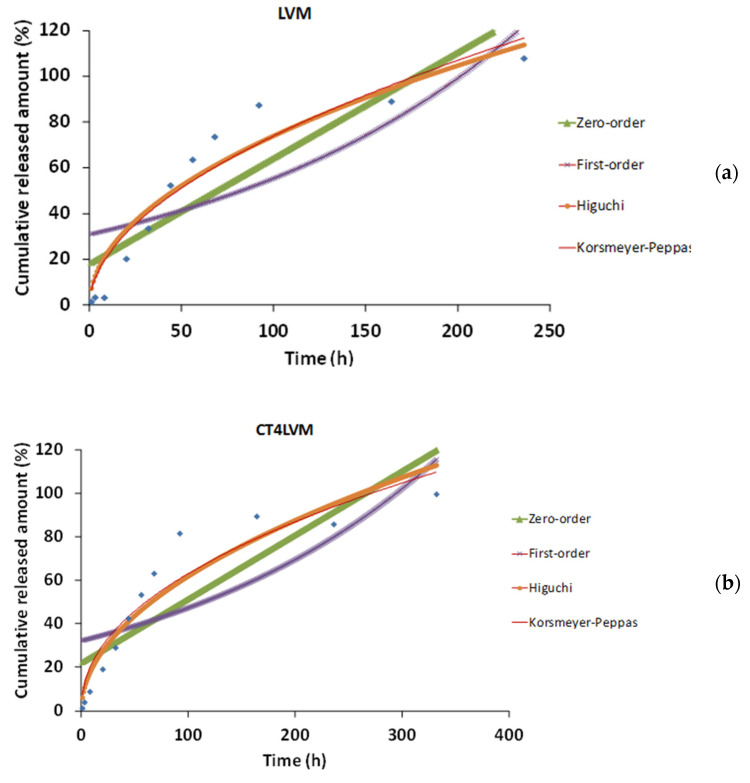

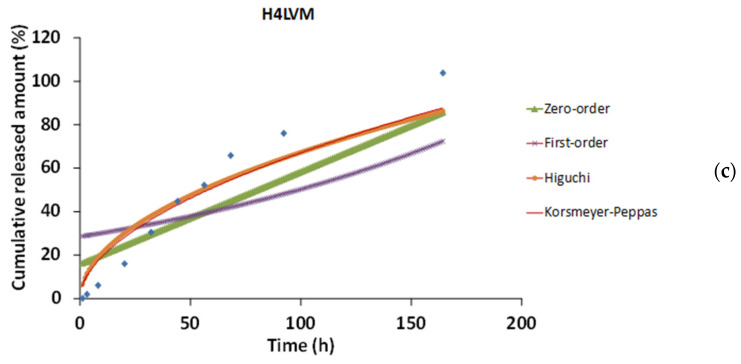
The mathematical models applied to the Curcumin release profile of LVM (**a**), CT4LVM (**b**), and H4LVM (**c**) (blue).

Figure 6 shows the theoretical release curves, obtained by applying the models, overlaid onto the experimental data, for each of the samples.

Table 3 displays the values of the parameters and the coefficients calculated with each of the four mathematical models for the delivery systems of the active ingredients.

For all systems, the lowest values for the adjusted correlation coefficients were obtained for the equation of the first-order model, followed by the zero-order one. Therefore, these two equations are not suitable for describing the release profile of the active principle from the studied colloidal vector system. Furthermore, Table 3 shows that the values of the adjusted correlation coefficients for the Korsmeyer-Peppas model are very close to those of the Higuchi model, which best explains the release profile of Curcumin in all three systems: LVM microemulsion, and gel microemulsion with chitosan or hyaluronic, respectively.

Some preview studies confirm that the Higuchi model best fits the release profile of the Curcumin from microemulsion [55,56]. According to the Higuchi model, the active principle is homogeneously dispersed and the release, from these three selected samples, is achieved by pure diffusion and without erosion or swelling of the matrix [57].

### 3.5. In Vitro Permeation Study

Because the proposed gel microemulsion formulations are being proposed as transdermal delivery systems, an in vitro permeation study was performed using a Strat-M^®^ membrane. The major drawbacks of the use of ex vivo human or animal skin in drug permeation studies include the complexity of preparation and storage of the specimens, the variability of the collected samples, and high cost. Synthetic membranes have been developed as an alternative to natural skin, and possess some important advantages, such as low cost, reduced space and simplicity of storage, and higher reproducibility of data. One of the synthetic membranes most studied in terms of correlation with human skin results is Strat-M^®^, a multilayered polymeric membrane, the morphology of which is designed to mimic the different layers of human skin. Recent literature has reported the similar permeability coefficients and partition parameters being obtained for many active pharmaceutical ingredients in permeation experiments with Strat-M^®^ membrane and human skin, and recommended the use of this synthetic membrane for in vitro screening tests for formulation optimization [59,60]. Additionally, since the novel formulation in the present study is proposed for application in the cosmetic industry, where the use of animals is banned in testing products, the Strat-M^®^ membrane was used in the permeation studies.

Three formulations were selected to be subjected to the permeation study, namely LVM, the parent microemulsion, and CT4LVM and H4LVM, the gel microemulsions with the maximum amounts of hyaluronic acid and chitosan, respectively. The cumulative drug permeation, steady-state fluxes, apparent permeability coefficients and retained Curcumin amounts in the Strat-M^®^ membrane were determined [26]. The steady-state fluxes and apparent permeability coefficients for Curcumin permeation through the model membrane from the microemulsion and the two gel microemulsions are shown in Table 4.

The permeability coefficient P_app_ was very high (2.64∙× 10^−3^ cm/h) for Curcumin from the simple microemulsion, and was higher than the value obtained for the other microemulsion formulations. The permeation decreased with gel microemulsions by up to 1.97 × 10^−3^ cm/h for the microemulsion with Chitosan and 1.16 × 10^−3^ cm/h for the microemulsion with hyaluronic acid, due to the viscosity changes in systems containing rather large amounts of polymers.

**Figure 7:**
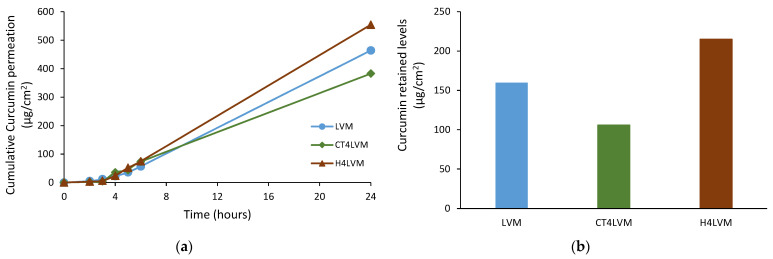
In vitro permeation of Curcumin from microemulsion and gel microemulsions through the Strat-M^®^ membrane (**a**) and the total Curcumin retained in the membrane after 24 h (**b**).

In Figure 7, the cumulative permeation over a period of 24 h and the total amount of Curcumin retained in the model membrane are presented.

For all three formulations, the amount of the permeated Curcumin Q_t_ increased in a time-dependent manner. These results are in concordance with previous studies, in which the permeation ability of various microemulsions incorporating hydrophobic active pharmaceutical ingredients was studied [31]. The value of Q_t_ at 24 h was about three times higher than the amount of Curcumin retained in the membrane; therefore, more drug is delivered transdermically than is expected to be retained in the skin. The amounts of Curcumin retained in the membrane were: 159.76 µg/cm^2^ for the LVM, 105.84 µg/cm^2^ for the CT4LVM and 214.82 µg/cm^2^ for the H4LVM. The highest cumulative permeation was obtained for the gel microemulsion with sodium hyaluronate (554.51 µg/cm^2^), while the lowest one was obtained for the gel microemulsion with chitosan (382.73 µg/cm^2^). The influence of the nature of polyelectrolyte on drug permeation is based on the diffusion patterns from matrixes with different viscosity values and the possible complexes between Curcumin molecules and polyelectrolyte chains with various charges.

Compared to the results reported in other papers on the permeability of Curcumin incorporated in microemulsion systems [31,35], the values for Q_t_ at 24 h and P_app_ in the case of the simple microemulsion LVM and the gel microemulsions CT4LVM and H4LVM forms were significantly higher, suggesting an improvement of the Curcumin transdermal permeability. The high permeation rate of these three formulations may be due to both the small droplet size and high concentration [28] of Curcumin, namely 1.1 wt.% in the LVM microemulsion; 1.6 wt.% in the CT4LVM and H4LVM gel microemulsions.

### 3.6. Biocompatibility

Biocompatibility is an essential characteristic for biomedical and cosmetic applications. Because the microemulsion and gel microemulsions developed in this study are intended to be applied topically, before performing any in vivo test it is necessary to determine the cytotoxicity potential in vitro. The biocompatibility of the microemulsion and gel microemulsions was evaluated and measured by MTT assay.

**Figure 8:**
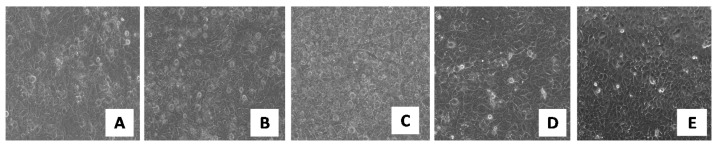
Keratinocytes visualized under a phase-contrast inverted light microscope (magnification 40×) after being treated with the following samples: (**A**) LVM; (**B**) CP3LVM; (**C**) H4LVM; (**D**) CT4LVM; (**E**) Control.

The assay results can be seen in Figure 8. A cell density of 100% was observed for the control (Figure 8E), while in the pictures containing the keratinocytes treated with the respective samples (Figure 8A–D), gaps can be observed (areas without texture), which are actually keratinocytes that are no longer viable.

The sample containing chitosan (Figure 8D) shows a cell viability of 80% and can be considered according to ISO 10993-5 to be non-toxic, unlike the rest of the samples (Figure 8A–C), which show a moderate toxicity.

**Figure 9:**
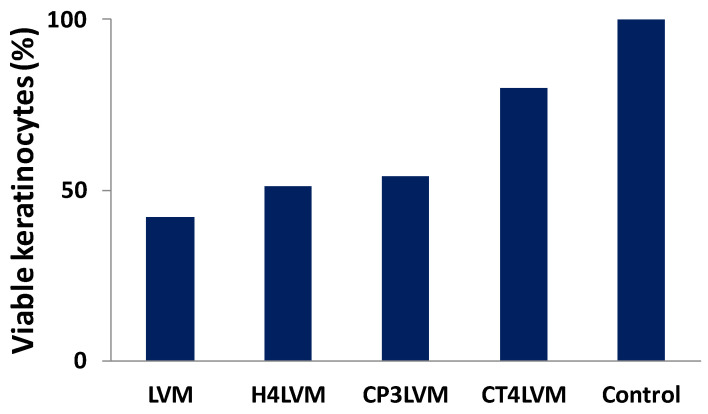
Percentage of viable keratinocytes for LVM microemulsion and gel microemulsions.

The biocompatibility characteristic can be observed (Figure 8 and Figure 9) to increase in the following order: LVM; H4LVM; CP3LVM; CT4LVM. The highest value of cell viability (80%) was obtained for the sample containing chitosan. The cell viability of approximately 50% for H4LVM and CP3LVM systems and approximately 40% for LVM was caused by the high concentrations of surfactant mixture required to stabilize the microemulsion. It is important to note that cytotoxicity tests, for which suspended cells are used, exaggerate adverse effects compared to in vivo cytotoxicity tests because they do not take into account the presence of the natural barrier of the protection of human skin and the continuous tissue repair processes of epithelial tissue [61,62]. According to several sources, the increase in the cell viability for the formulation with chitosan may be due to the ability of chitosan to increase cell viability and the degree of keratinocyte proliferation [63,64]. The characteristic whereby chitosan is able to improve the penetration into the skin of formulations in which it is included represents an important advantage for topical applications [61]. Thus, gel microemulsions, especially those with chitosan, are better suited for topical applications, compared to the simple LVM microemulsion.

## 4. Conclusions

The purpose of this work was to obtain and characterize novel gel microemulsions that could be used for topical cosmetic applications, using grape seed oil as an oily phase. It was also proposed to conduct a study to evaluate the capacity of the newly obtained systems, namely microemulsion and gel microemulsions, to encapsulate and release Curcumin.

Winsor IV microemulsion was obtained using biocompatible surfactants, at a minimum concentration, with a suitable balance of surfactant-cosurfactant to ensure the stability of the system. The obtained microemulsion was further used as the base for gel microemulsion carriers, prepared using Carbopol^®^ 980 NF as a synthetic polymer, and two polymers of natural origin, chitosan and sodium hyaluronate. The physico-chemical characterization step of the obtained microemulsion was necessary to determine its structure and to establish the type of polymers suitable for obtaining gel microemulsions.

In this study, an important amount of Curcumin was encapsulated in the optimized microemulsion and gel microemulsions; approximately 1.7 (*w/w*)%.

The gel microemulsions exhibited a pseudoplastic non-Newtonian rheological behavior. At a similar mass concentration of thickening agent, Carbopol^®^ 980 NF showed a lower viscosity value compared to the other two polymers. In the case of the gel microemulsions with hyaluronic acid and those with chitosan, close values of viscosity were obtained.

The evaluation of cell viability for keratinocytes as a normal cells model showed that the gel microemulsions had a lower cytotoxicity than the simple microemulsion, and for the gel microemulsions, the percentage of viable keratinocytes increased in the order hyaluronic acid, Carbopol^®^ 980 NF, then chitosan.

Taking into account the fact that the highest viscosity was obtained for gel microemulsions with chitosan, and their high biocompatibility with the skin, the CT4LVM sample would be the most suitable as a system for the delivery and controlled release of active ingredients in the skin.

Due to the physico-chemical characteristics of gel microemulsions, high encapsulation capacities and the improvement of the penetration degree of the active principles, these systems are suitable to be used as colloidal vectors for formulations in topical application of Curcumin in dermatocosmetic products.

## Figures and Tables

**Table 1:** Polymer content of the gel microemulsions prepared.

Sample	Polymer	Polymer Content ^1^ wt.%
H1LVM	Hyaluronic acid Na salt	0.1
H2LVM	Hyaluronic acid Na salt	0.15
H3LVM	Hyaluronic acid Na salt	0.2
H4LVM	Hyaluronic acid Na salt	0.3
CP1LVM	Carbopol^®^ 980 NF	0.1
CP2LVM	Carbopol^®^ 980 NF	0.15
CP3LVM	Carbopol^®^ 980 NF	0.2
CT1LVM	Chitosan	0.115
CT2LVM	Chitosan	0.23
CT3LVM	Chitosan	0.298
CT4LVM	Chitosan	0.34

^1^ Based on the entire microemulsion mass.

**Table 2:** Appearance and composition of the LVM microemulsion.

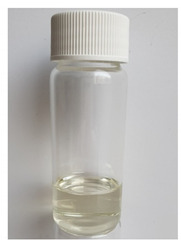	**Ingredients**	**Function**	**Mass Concentration %**
	Grape seed oil	Oily phase	7.6
	Water	Aqueous phase	23.7
	Plurol^®^ Diisostearique CG	Surfactant	15.1
	Tween 80	Surfactant	45.2
	Ethanol	Co-solvent	8.4

**Table 3:** Parameters of each model applied to the selected samples to study the mechanism of Curcumin release.

Model	Parameter	Formulation		
		LVM	CT4LVM	H4LVM
Zero-order ^1^ Q_t_ = Q_0_ + K_0_ ∙ t	K_0_	0.462	0.294	0.425
	Q_0_	18.378	22.254	16.071
	R^2^_adj_	0.730	0.678	0.697
First-order ^1^ ln Q_t_ = ln Q_0_ + K_1th_ ∙ t	K_1th_	0.005	0.003	0.005
	Q_0_	31.008	32.322	28.660
	R^2^_adj_	0.533	0.503	0.468
Higuchi ^1^ Q_t_ = Q_0_ + K_H_ ∙ t^1/2^	K_H_	7.417	6.199	6.748
	Q_0_	0	0	0
	R^2^_adj_	0.896	0.887	0.864
Korsmeyer-Peppas ^1^ Q_t_ = Q_0_+ K_KP_ ∙ t^n^	K_KP_	6.370	7.533	5.555
	Q_0_	0	0	0
	n	0.532	0.461	0.541
	R^2^_adj_	0.884	0.878	0.846

^1^ where “K” is the kinetic constant, “Q_t_” is the cumulative amount of drug released at time “t”, and “Q_0_” is the initial amount of drug; for Korsmeyer-Peppas equation “Q_0_” is the amount of the drug released after infinite time [52,53,54].

**Table 4:** In vitro permeability parameters: steady-state fluxes, and apparent permeability coefficients of Curcumin loaded in microemulsion and gel microemulsions.

Sample	Time (h)	J_ss_ (µg/cm^2^∙h)	P_app_ (10^−3^ cm/h)
LVM	4	28.59	2.64
	5	13.26	1.22
	6	21.30	1.97
	24	22.86	2.11
CT4LVM	4	33.22	1.97
	5	8.19	0.49
	6	29.99	1.78
	24	17.38	1.03
H4LVM	4	18.60	1.16
	5	28.17	1.75
	6	22.94	1.43
	24	26.93	1.68

## Data Availability

Not applicable.

## References

[1] Grampurohit N., Ravikumar P., Mallya R. (2009). Microemulsions for topical use–A review. Indian J. Pharm. Educ. Res..

[2] Asahi M., Shirakawa N.E.M.Y., Kikuta Y. (1993). Water-in-Oil Type Emulsion Cosmetic. EU. Patent.

[3] Kogan A., Garti N. (2006). Microemulsions as transdermal drug delivery vehicles. Adv. Colloid Interface Sci..

[4] Aggarwal N., Goindi S., Khurana R. (2013). Formulation, characterization and evaluation of an optimized microemulsion formulation of griseofulvin for topical application. Colloids Surf. B Biointerfaces.

[5] Patel M.R., Patel R.B., Parikh J.R., Patel B.G. (2016). Novel microemulsion-based gel formulation of tazarotene for therapy of acne. Pharm. Dev. Technol..

[6] Tadros T.F. (1992). Future developments in cosmetic formulations. Int. J. Cosmet. Sci..

[7] Ali J., Akhtar N., Sultana Y., Baboota S., Ahuja A. (2008). Antipsoriatic microemulsion gel formulations for topical drug delivery of Babchi oil (Psoralea Corylifolia). Methods Find. Exp. Clin. Pharmacol..

[8] Surini S., Mellani T. (2017). Formulation and physical evaluation of microemulsion and W/O/W multiple emulsions dosage forms with alpha arbutin, lactic acid, and niacinamide as skin-whitening cosmetics. Int. J. Appl. Pharm..

[9] Boonme P., Junyaprasert V.B. (2010). Characterization and stability of vitamin A palmitate microemulsions composed of Isopropyl Palmitate, Water and Polyoxyethylene-10-Oleyl Ether. J. Pharm. Sci..

[10] Roohinejad S., Oey I., Wen J., Lee S.J., Everett D.W., Burritt D.J. (2015). Formulation of oil-in-water β-Carotene microemulsions: Effect of oil type and fatty acid chain length. Food Chem..

[11] Azeem A., Rizwan M., Ahmad F., Khan Z., Khar R., Aqil M., Talegaonkar S. (2008). Emerging role of microemulsions in cosmetics. DDF.

[12] Schreiber J., Wolf F., Croizet D. (2002). Cosmetic or Pharmaceutical Lecithin-Containing Gels or Low Viscosity Lecithin-Containing O/W Microemulsions. U.S. Patent.

[13] Mehta D.P., Rathod H.J., Shah D.P., Shah C.N. (2015). A review on microemulsion based gel: A recent approach for topical drug delivery system. Res. J. Pharm. Technol..

[14] Patel R.R., Rahul R., Kanu R.P., Mukesh R.P. (2014). Formulation and characterization of microemulsion based gel of antifungal drug. PharmaTutor.

[15] Chhatrani B.M., Shah D.D.P. (2017). A review on microemulsion based gel: A novel approach for enhancing topical delivery of hydrophobic drug. Int. J. Pharm. Pharm. Res..

[16] Ghosh M., Kundu S., Pyne A., Sarkar N. (2020). Unveiling the behavior of curcumin in biocompatible microemulsion and its differential interaction with gold and silver nanoparticles. J. Phys. Chem. C.

[17] Jadhav B.-K., Mahadik K.-R., Paradkar A.-R. (2007). Development and validation of improved reversed phase-HPLC method for simultaneous determination of Curcumin, Demethoxycurcumin and Bis-Demethoxycurcumin. Chroma.

[18] Gopinath D., Ahmed M.R., Gomathi K., Chitra K., Sehgal P.K., Jayakumar R. (2004). Dermal wound healing processes with curcumin incorporated collagen films. Biomaterials.

[19] Moniruzzaman M., Min T. (2020). Curcumin, curcumin nanoparticles and curcumin nanospheres: A review on their pharmacodynamics based on monogastric farm animal, poultry and fish nutrition. Pharmaceutics.

[20] Kaur T., Kapoor D. (2018). Development and evaluation of sea buckthorn (Hippophae Rhamnoides, L.) seed oil nanoemulsion gel for wound healing. Phcog. Mag..

[21] Kupper S., Kłosowska-Chomiczewska I., Szumała P. (2017). Collagen and hyaluronic acid hydrogel in water-in-oil microemulsion delivery systems. Carbohydr. Polym..

[22] Papakonstantinou E., Roth M., Karakiulakis G. (2012). Hyaluronic acid: A key molecule in skin aging. Derm. Endocrinol..

[23] Ahmadi R., de Bruijn J.D. (2008). Biocompatibility and gelation of chitosan–glycerol phosphate hydrogels. J. Biomed. Mater. Res..

[24] Yousef S.A., Mohammed Y.H., Namjoshi S., Grice J.E., Benson H.A.E., Sakran W., Roberts M.S. (2019). Mechanistic evaluation of enhanced curcumin delivery through human skin in vitro from optimised nanoemulsion formulations fabricated with different penetration enhancers. Pharmaceutics.

[25] Lin H.-Y., Thomas J.L., Chen H.-W., Shen C.-M., Yang W.-J., Lee M.-H. (2012). In vitro suppression of oral squamous cell carcinoma growth by ultrasound-mediated delivery of curcumin microemulsions. Int. J. Nanomed..

[26] Lin C.-C., Lin H.-Y., Chen H.-C., Yu M.-W., Lee M.-H. (2009). Stability and characterisation of phospholipid-based curcumin-encapsulated microemulsions. Food Chem..

[27] Sintov A.C. (2015). Transdermal delivery of curcumin via microemulsion. Int. J. Pharm..

[28] Liu C.-H., Chang F.-Y., Hung D.-K. (2011). Terpene microemulsions for transdermal curcumin delivery: Effects of terpenes and cosurfactants. Colloids Surf. B: Biointerfaces.

[29] Scomoroscenco C., Cinteza L.O., Teodorescu M., Gifu I.C., Ianchis R., Nistor C.L., Petcu C., Ninciuleanu C.M., Alexandrescu E., Mihaescu C.I. (2020). Vegetable oil-based microemulsions with dermato-cosmetic applications. U.P.B. Sci. Bull. Ser. B.

[30] Froelich A., Osmałek T., Snela A., Kunstman P., Jadach B., Olejniczak M., Roszak G., Białas W. (2017). Novel microemulsion-based gels for topical delivery of indomethacin: Formulation, physicochemical properties and in vitro drug release studies. J. Colloid Interface Sci..

[31] Kale S.N., Deore S.L. (2016). Emulsion micro emulsion and nano emulsion: A review. SRP.

[32] Gharbavi M., Manjili H.K., Amani J., Sharafi A., Danafar H. (2019). In vivo and in vitro biocompatibility study of novel microemulsion hybridized with bovine serum albumin as nanocarrier for drug delivery. Heliyon.

[33] Wang L.-L., Huang S., Guo H., Han Y., Zheng W., Jiang J. (2016). In situ delivery of thermosensitive gel-mediated 5-Fluorouracil microemulsion for the treatment of colorectal cancer. DDDT.

[34] Badawi A.A., Nour S.A., Sakran W.S., El-Mancy S.M.S. (2009). Preparation and evaluation of microemulsion systems containing salicylic acid. AAPS Pharm. Sci. Tech..

[35] Krauel K., Girvan L., Hook S., Rades T. (2007). Characterisation of colloidal drug delivery systems from the naked eye to Cryo-FESEM. Micron.

[36] Shinde U., Pokharkar S., Modani S. (2012). Design and evaluation of microemulsion gel system of nadifloxacin. Indian J. Pharm. Sci..

[37] Baboota S., Sharma S., Kumar A., Alam M.S., Sahni J., Ali J. (2011). Nanocarrier-based hydrogel of betamethasone dipropionate and salicylic acid for treatment of psoriasis. Int. J. Pharma. Investig..

[38] Parente M.E., Ochoa Andrade A., Ares G., Russo F., Jiménez-Kairuz Á. (2015). Bioadhesive hydrogels for cosmetic applications. Int. J. Cosmet. Sci..

[39] Bergonzi M.C., Hamdouch R., Mazzacuva F., Isacchi B., Bilia A.R. (2014). Optimization, characterization and in vitro evaluation of curcumin microemulsions. LWT Food Sci. Technol..

[40] Tønnesen H.H., Másson M., Loftsson T. (2002). Studies of curcumin and curcuminoids. XXVII. Cyclodextrin complexation: Solubility, chemical and photochemical stability. Int. J. Pharm..

[41] Fonseca-Santos B., Gremião M.P.D., Chorilli M. (2017). A simple reversed phase high-performance liquid chromatography (HPLC) method for determination of in situ gelling curcumin-loaded liquid crystals in in vitro performance tests. Arab. J. Chem..

[42] Khor P.Y., Mohd Aluwi M.F.F., Rullah K., Lam K.W. (2019). Insights on the synthesis of asymmetric curcumin derivatives and their biological activities. Eur. J. Med. Chem..

[43] Sharma S., Ganju E., Upmanyu N., Jain P. (2018). Therapeutic microemulsion of curcumin for the management of osteoarthritis. J. Drug Deliv. Ther..

[44] Shin G.H., Li J., Cho J.H., Kim J.T., Park H.J. (2016). Enhancement of curcumin solubility by phase change from crystalline to amorphous in cur-TPGS nanosuspension. J. Food Sci..

[45] Takenaka M., Ohkubo T., Okadome H., Sotome I., Itoh T., Isobe S. (2013). Effective extraction of curcuminoids by grinding turmeric (Curcuma Longa) with medium-chain Triacylglycerols. FSTR.

[46] Xu J., Xu B., Shou D., Xia X., Hu Y. (2015). Preparation and evaluation of vancomycin-loaded N-Trimethyl chitosan nanoparticles. Polymers.

[47] Ravikumar P., Tatke P. (2019). Design of an encapsulated topical formulation for chemoprevention of skin cancer. IJPSR.

[48] Liu C.-H., Chang F.-Y. (2011). Development and characterization of Eucalyptol microemulsions for topic delivery of curcumin. Chem. Pharm. Bull..

[49] Wang S., Chen P., Zhang L., Yang C., Zhai G. (2012). Formulation and evaluation of microemulsion-based in situ ion-sensitive gelling systems for intranasal administration of curcumin. J. Drug Target..

[50] Setthacheewakul S., Mahattanadul S., Phadoongsombut N., Pichayakorn W., Wiwattanapatapee R. (2010). Development and evaluation of self-microemulsifying liquid and pellet formulations of curcumin, and absorption studies in rats. Eur. J. Pharm. Biopharm..

[51] Kuang J., Gao J., Xie S., Lei Q., Fang W., Xie H., Lu X. (2020). Phase behaviors and curcumin encapsulation performance of gemini surfactant microemulsion. J. Mol. Liq..

[52] Cojocaru V., Ranetti A.E., Hinescu L.G., Ionescu M., Cosmescu C., Po A.G., Cintez L.O. (2015). Formulation and evaluation of in vitro release kinetics of Na3CaDTPA decorporation agent embedded in microemulsion-based gel formulation for topical delivery. Farmacia.

[53] Costa P., Sousa Lobo J.M. (2003). Evaluation of mathematical models describing drug release from estradiol transdermal systems. Drug Dev. Ind. Pharm..

[54] Baishya H. (2017). Application of mathematical models in drug release kinetics of carbidopa and levodopa ER tablets. J. Dev. Drugs.

[55] Zainuddin N., Ahmad I., Zulfakar M.H., Kargarzadeh H., Ramli S. (2021). Cetyltrimethylammonium bromide-nanocrystalline cellulose (CTAB-NCC) based microemulsions for enhancement of topical delivery of curcumin. Carbohydr. Polym..

[56] Shukla T., Upmanyu N., Agrawal M., Saraf S., Saraf S., Alexander A. (2018). Biomedical applications of microemulsion through dermal and transdermal route. Biomed. Pharmacother..

[57] Kobryń J., Zięba T., Sowa S.K., Musiał W. (2020). Influence of acetylated annealed starch on the release of β-escin from the anionic and non-ionic hydrophilic gels. Pharmaceutics.

[58] Forouz F., Dabbaghi M., Namjoshi S., Mohammed Y., Roberts M.S., Grice J.E. (2020). Development of an oil-in-water self-emulsifying microemulsion for cutaneous delivery of Rose Bengal: Investigation of anti-melanoma properties. Pharmaceutics.

[59] Haq A., Goodyear B., Ameen D., Joshi V., Michniak-Kohn B. (2018). Strat-M^®^ synthetic membrane: Permeability comparison to human cadaver skin. Int. J. Pharm..

[60] Bolla P.K., Clark B.A., Juluri A., Cheruvu H.S., Renukuntla J. (2020). Evaluation of formulation parameters on permeation of Ibuprofen from topical formulations using Strat-M^®^ membrane. Pharmaceutics.

[61] Chen H., Chang X., Du D., Li J., Xu H., Yang X. (2006). Microemulsion-based hydrogel formulation of ibuprofen for topical delivery. Int. J. Pharm..

[62] Contri R.V., Fiel L.A., Alnasif N., Pohlmann A.R., Guterres S.S., Schäfer-Korting M. (2016). Skin penetration and dermal tolerability of acrylic nanocapsules: Influence of the surface charge and a chitosan gel used as vehicle. Int. J. Pharm..

[63] Howling G.I., Dettmar P.W., Goddard P.A., Hampson F.C., Dornish M., Wood E.J. (2001). The effect of chitin and chitosan on the proliferation of human skin fibroblasts and keratinocytes in vitro. Biomaterials.

[64] López-García J., Lehocký M., Humpolíček P., Sáha P. (2014). HaCaT keratinocytes response on antimicrobial atelocollagen substrates: Extent of cytotoxicity, cell viability and proliferation. J. Funct. Biomater..

